# AML-VAC-XS15-01: protocol of a first-in-human clinical trial to evaluate the safety, tolerability and preliminary efficacy of a multi-peptide vaccine based on leukemia stem cell antigens in acute myeloid leukemia patients

**DOI:** 10.3389/fonc.2024.1458449

**Published:** 2024-10-14

**Authors:** Susanne Jung, Annika Nelde, Yacine Maringer, Monika Denk, Lisa Zieschang, Christine Kammer, Melek Özbek, Peter Martus, Christopher Hackenbruch, Alexander Englisch, Jonas S. Heitmann, Helmut R. Salih, Juliane S. Walz

**Affiliations:** ^1^ Clinical Collaboration Unit Translational Immunology, German Cancer Consortium (DKTK), Department of Internal Medicine, University Hospital Tübingen, Tübingen, Germany; ^2^ Department of Peptide-based Immunotherapy, Institute of Immunology, University of Tübingen and University Hospital Tübingen, Tübingen, Germany; ^3^ Cluster of Excellence iFIT (EXC2180) ‘Image-Guided and Functionally Instructed Tumor Therapies’, University of Tübingen, Tübingen, Germany; ^4^ German Cancer Consortium (DKTK) and German Cancer Research Center (DKFZ), Partner Site Tübingen, Tübingen, Germany; ^5^ Institute for Clinical Epidemiology and Applied Biometry, University and Faculty of Medicine, University of Tübingen, Tübingen, Germany; ^6^ Department of Women’s Health, University Hospital Tübingen, Tübingen, Germany

**Keywords:** acute myeloid leukemia, peptide vaccine, immunotherapy, clinical trial, translational immunology

## Abstract

**Introduction:**

Acute myeloid leukemia (AML) has a dismal prognosis, mostly due to minimal residual disease-driven relapse, making an elimination of persisting therapy-resistant leukemia progenitor/stem cells (LPCs) the main goal for novel therapies. Peptide-based immunotherapy offers a low-side-effect approach aiming to induce T cell responses directed against human leukocyte antigen (HLA) presented tumor antigens on malignant cells by therapeutic vaccination. Mass spectrometry-based analysis of the naturally presented immunopeptidome of primary enriched LPC and AML samples enabled the selection of antigens exclusively expressed on LPC/AML cells, which showed *de novo* induction and spontaneous memory T cell responses in AML patients, and whose presentation and memory T cell recognition was associated with improved disease outcome.

**Methods:**

Based on these data the therapeutic vaccine AML-VAC-XS15 was designed, comprising two mutated HLA class I-restricted peptides from the common AML-specific mutation in NPM1 and seven HLA class II-restricted peptides (six non-mutated high-frequent AML/LPC-associated antigens and one mutated peptide from the AML-specific mutation R140Q in IDH2), adjuvanted with the toll like receptor 1/2 ligand XS15 and emulsified in Montanide ISA 51 VG. A phase I open label clinical trial investigating AML-VAC-XS15 was designed, recruiting AML patients in complete cytological remission (CR) or CR with incomplete blood count recovery. Patients are vaccinated twice with a six-week interval, with an optional booster vaccination four months after 2nd vaccination, and are then followed up for two years. The trial’s primary objectives are the assessment of the vaccine’s immunogenicity, safety and toxicity, secondary objectives include characterization of vaccine-induced T cell responses and assessment of preliminary clinical efficacy.

**Ethics and dissemination:**

The AML-VAC-XS15-01 study was approved by the Ethics Committee of the Bavarian State medical association and the Paul-Ehrlich Institut (P01392). Clinical trial results will be published in peer-reviewed journals.

## 1 Introduction

Acute myeloid leukemia (AML), a malignant disorder of the bone marrow characterized by the clonal expansion and differentiation arrest of myeloid progenitor cells (1), is the most common form of acute leukemia in adults, yet shows the worst outcome of all leukemias with a 5-year survival rate of only 30% (2). Despite numerous treatment advances in recent years and high initial remission rates, disease relapse occurs in a large proportion of patients, resulting in the still very high mortality rate of AML (3). Most treatment advances that were achieved over the last decades have mainly led to a benefit for younger patients, as they tend to be based on intensive chemotherapy and the increased use of allogeneic stem cell transplantation. Moreover, attempts to find treatment strategies based on the increasing knowledge on the molecular subgroups of AML have led to the approval of a number of new substances like enasidenib, ivosidenib or gilteritinib. However, none of them have so far shown a significant impact on overall survival (4). The prognosis for medically unfit patients and patients above the age of seventy remains dismal due to their ineligibility for intensive treatment and the resulting high relapse rates (5, 6). The main reason for disease relapse is the presence of minimal residual disease (MRD), which is characterized by the persistence of therapy-resistant leukemia stem and progenitor cells (LPCs) in the patients’ bone marrow (7). To eliminate these cells and achieve long-lasting remissions and potential cure, the development of novel therapeutic approaches that specifically target these residual cells is urgently needed.

One option to achieve this goal is T cell-based immunotherapy, which harnesses the immune system to eliminate malignant cells. The immunogenicity of AML, which is documented by graft-versus-leukemia effects after hematopoietic stem cell transplantation (8) as well as the favorable immune effector-to-target cell ratios in the MRD setting, suggests that AML might be effectively targeted by T cell-based immunotherapy (9). In recent years, the breakthrough clinical success of T cell-based immunotherapy approaches, including immune checkpoint inhibitors, chimeric antigen receptor T cells, bispecific antibodies and adoptive T cell transfer, have revolutionized the treatment of many malignant diseases (10–12). However, the majority of patients and cancer entities, including AML, still do not benefit from available immunotherapeutic strategies, others for a limited time only (13–16). Remaining challenges to augment the efficacy of T cell-based immunotherapy are therefore to improve specificity of treatment, to increase the frequency of anti-tumor immune responses, as well as to reduce toxicity and to expand the spectrum of targetable cancer entities. A rational and promising low side-effect approach to achieve this goal is peptide-based immunotherapy (17, 18), which aims to induce a T cell response directed against tumor antigens (TAs), presented on the surface of tumor cells via human leukocyte antigen (HLA) molecules, by using therapeutic vaccination (19). These TAs can arise from tumor-specific mutations or from non-mutated, differentially expressed and presented gene and protein products in the tumor cell (19).

For the development of novel immunotherapeutic approaches for AML, the specific therapeutic targeting of the relapse-inducing LPC subpopulation is of central importance. To identify widely applicable and naturally presented LPC antigens, we used a well-established mass spectrometry-based immunopeptidomics workflow within a large cohort of primary AML samples (taken from patients at first diagnosis as well as in a relapse situation), to identify frequent HLA class I- and HLA class II-restricted antigenic peptides presented exclusively on the CD34^+^CD38^-^ LPC subpopulation or on both LPCs and AML blasts (20). By comparative immunopeptidome profiling using a large and diverse benign immunopeptidome database (20–22), antigens were selected based on their frequent and exclusive presentation within the AML cohort, with no detection on any benign tissue of hematologic or non-hematologic origin (23). Additionally, screening of the AML/LPC immunopeptidomes for peptides derived from common AML-specific mutations (*e.g.* FLT3, NPM1, IDH2, DNMT3A) enabled the identification of naturally presented neoepitopes of NPM1 and IDH2. Functional characterization of these novel mutated and non-mutated AML/LPC antigen targets showed *de novo* induction and spontaneous memory T cell responses in samples of AML patients and healthy volunteers (23). Of note, it was further shown that a diverse presentation of these AML-exclusive antigens is associated with an improved disease outcome in AML patients (23), highlighting the clinical relevance of these antigens.

Based on these analyses, an AML/LPC peptide vaccine was designed, comprising nine mutated and non-mutated AML- and AML/LPC-associated HLA class I and HLA class II-restricted peptides. As an adjuvant our toll like receptor (TLR) 1/2 agonist XS15 (24) emulsified in Montanide ISA 51 VG was chosen, which has already been used in several clinical trials (25–27), showing a beneficial safety profile as well as the induction of strong and long-lasting T cell responses even in immunocompromised cancer patients (19, 25, 26, 28). The first-in-human (FIH) study described here has been designed in accordance with the recommendations of the SPIRIT 2013 Statement (29) to evaluate the resulting multi-peptide vaccine AML-VAC-XS15, with regard to overall safety and tolerability, as well as first signs of clinical efficacy in AML patients.

## 2 Materials

**Figure 1:**
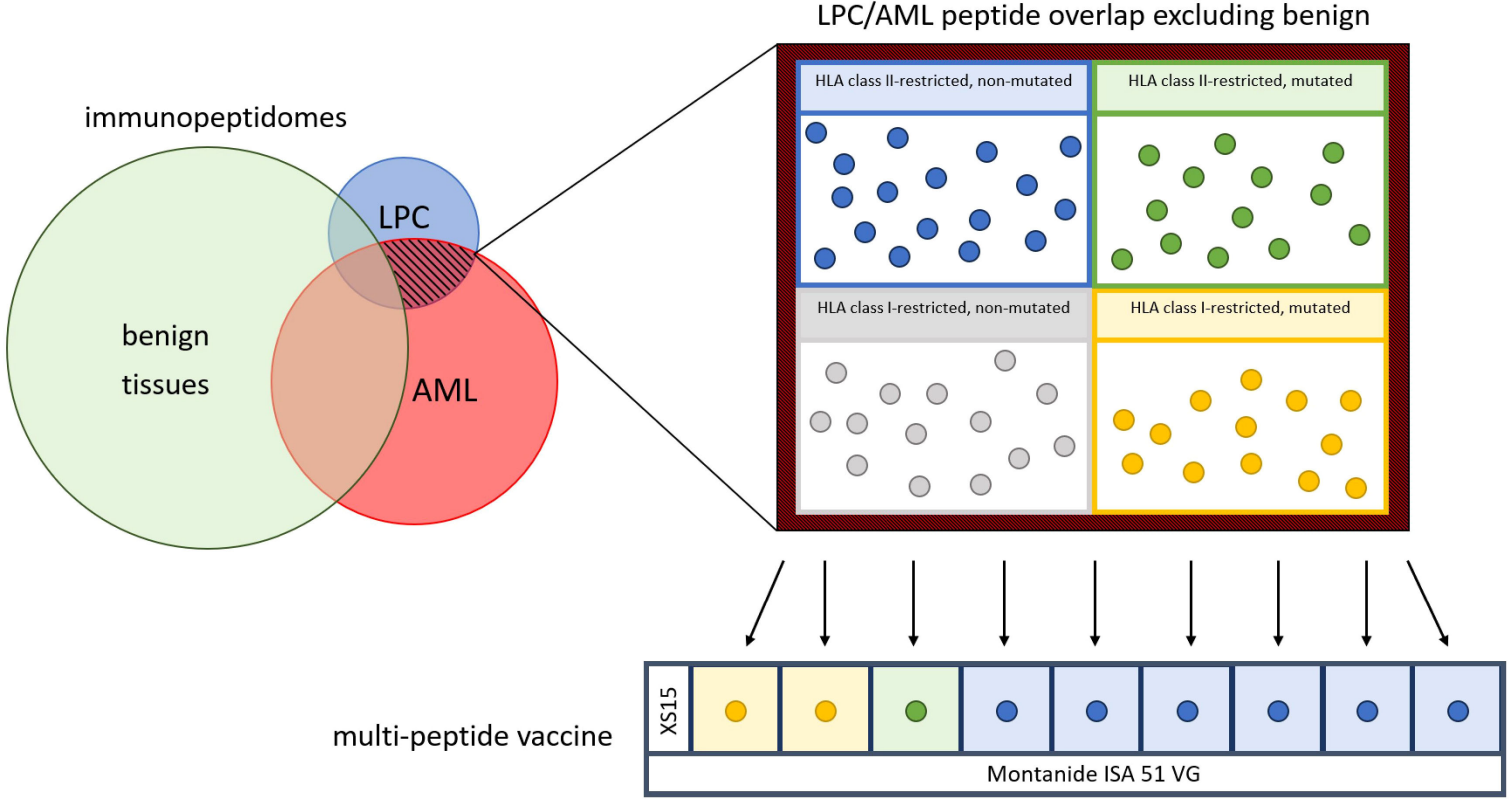
Construction of the vaccine. After an extensive comparison of immunopeptidomes of acute myeloid leukemia (AML) cells (red), leukemia stem and progenitor cells (LPC) (blue) and benign tissue cells (green), the LPC/AML non-benign overlap (red-black hatched) was isolated and used to provide six human leukocyte antigen (HLA) class II-restricted non-mutated (blue dots), one HLA class II-restricted mutated (green dot) and two HLA class I-restricted mutated (yellow dots) peptides for the peptide mix, including the adjuvant XS15. The peptide mix is emulsified with Montanide ISA 51 VG to formulate the multi-peptide vaccine.

The vaccine AML-VAC-XS15 evaluated in this trial consists of nine AML-specific peptides, the adjuvant XS15 and the emulsifier Montanide ISA 51 VG (**Figure 1**). The peptide mixture contains 300 µg each of one AML-specific mutated (R140Q in IDH2) peptide and six immunopeptidome-defined non-mutated AML- and LPC-associated HLA class II-restricted peptides with promiscuous binding to multiple HLA class II allotypes, as well as 300 µg each of two immunopeptidome-defined mutation-derived AML-associated HLA class I-restricted neoepitopes from the common AML-specific mutation in NPM1. All peptides were selected from a collection of AML- and AML/LPC-associated antigens characterized on primary AML blasts and LPCs by direct isolation of naturally presented HLA class I and HLA class II ligands from leukemia cells and subsequent identification via mass spectrometry. The peptides had been functionally characterized regarding their ability to induce T cell responses and their clinical relevance (23).

The lipopeptide XS15, chemical name N-Palmitoyl-S-[2,3-bis(palmitoyloxy)-(2R)-propyl]-Icysteinyl-GDPKHPKSF, is a water-soluble synthetic Pam3Cys-derivative. Due to its function as TLR1/2 ligand, it was chosen as a suitable adjuvant to complement the peptide cocktail in the AML-VAC-XS15-01 trial. Peptide mix and 0.12 mg XS15 are reconstituted in 33% dimethyl sulfoxide (DMSO)/water for injection.

Prior to application, the cocktail of AML-associated peptides and XS15 is emulgated in a water-oil emulsion 1:1 with Montanide ISA 51 VG. Montanide ISA 51 VG is based on a blend of mannide monooleate surfactant and mineral oil, has been used as an adjuvant in more than 200 human vaccine trials and renders stable water-in-oil emulsions when mixed with water-based antigenic media.

## 3 Methods

### 3.1 Participants

**Figure 2:**
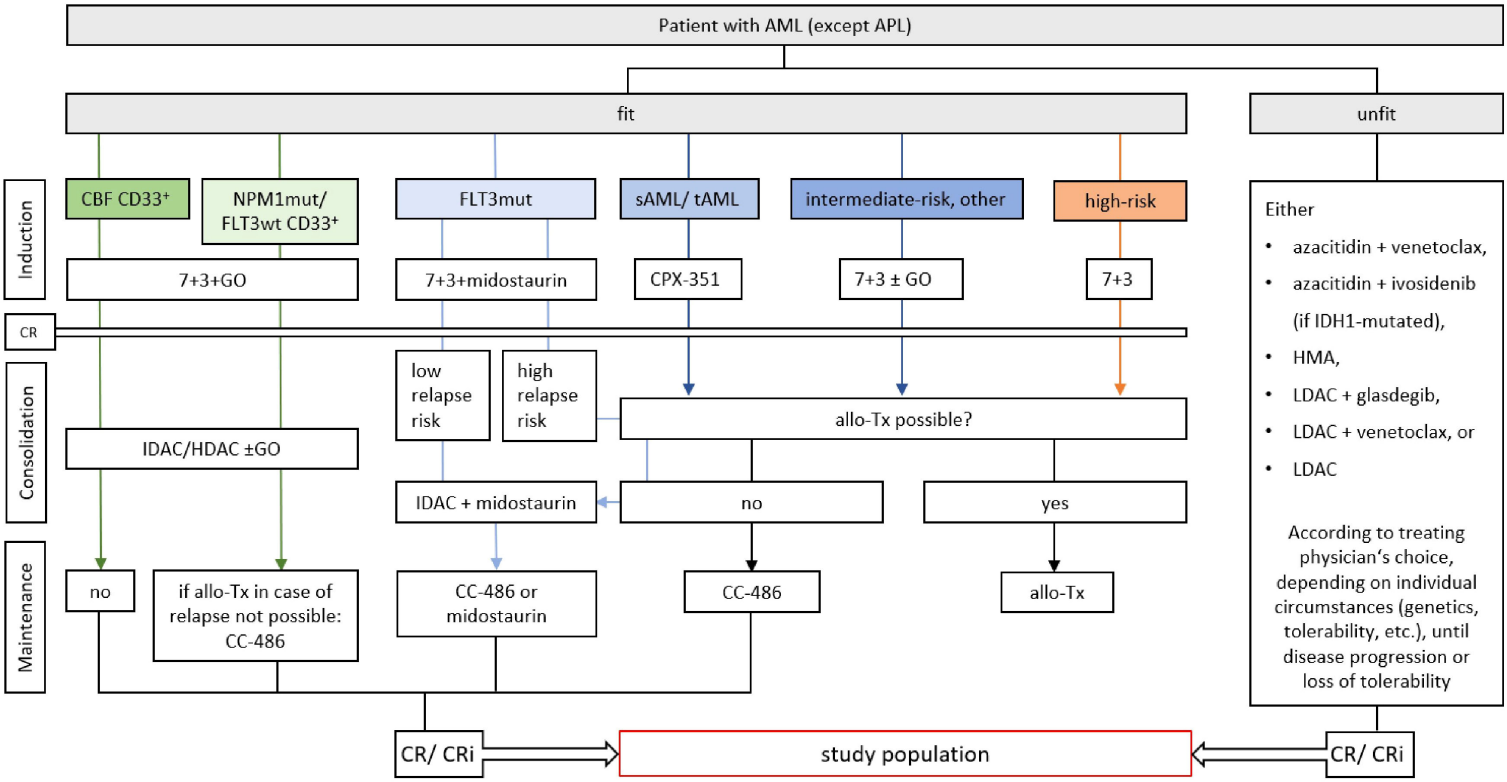
Study population within the framework of current treatment guidelines of the medical societies in hematology and medical oncology of the German speaking countries (DGHO) (4). Study patients must have reached complete remission (CR) or CR with incomplete blood count recovery (CRi) with any type of first-line therapy. Allogeneic stem cell transplantation (allo-Tx) must not be a viable treatment option. AML, acute myeloid leukemia; APL, acute promyelocyte leukemia; 7 + 3, combinatorial treatment with cytarabine (7 days) and daunorubicin (3 days); CBF, core binding factor mutation; GO, gemtuzumab ozogamicin; mut, mutated; sAML, secondary AML; tAML, therapy-associated AML; LDAC, low-dose cytarabine; IDAC, intermediate-dose cytarabine; HDAC, high-dose cytarabine; CC-486, oral azacytidine; CPX-352, liposomal cytarabine and daunorubicin; HMA, hypomethylating agent.

The trial enrolls 20 AML patients who have achieved complete remission (CR) or CR with incomplete blood count recovery (CRi) with first line therapy and are not eligible for an allogeneic stem cell transplantation (**Figure 2**). This includes AML patients who will continue to receive low-dose or maintenance therapy during the trial, with ongoing therapy being coordinated by the primary responsible oncologist. Patients must have achieved cytological CR/CRi but may be MRD positive or negative. By defining the study population in this fashion, adult patients from all age and fitness categories may be included, independent of risk classification or ongoing therapy. Only patients who are eligible for allogeneic stem cell transplantation are excluded, as in the course of an allogeneic stem cell transplantation the complete T cell compartment is replaced by the donor’s immune system. T cells of the patient are specifically destroyed as part of graft rejection and GvHD prophylaxis, T cells induced by the trial vaccine will thus be eliminated and replaced by donor cells without any effect of the vaccination being expected to remain. There will be no healthy control subjects in the study. Detailed inclusion and exclusion criteria are therefore as follows:

Inclusion criteria

- Ability to understand and willingness to sign a written informed consent document.
- Males or females aged ≥ 18 years of age.
- Documented diagnosis of AML according to WHO guidelines.
  o Morphological CR or CRi, positive MRD is permitted.
  o Completion of previous intensive therapy (first vaccination 4-28 weeks after last application of intensive chemotherapy).
  o Ongoing low intensity treatment, e.g. with hypomethylating agents (HMA) and venetoclax, is permitted.
  o Ongoing maintenance treatment, e.g. with oral azacytidine or midostaurin, is permitted.
  o Not eligible for allogeneic stem cell transplantation.
- Eastern Cooperative Oncology Group (ECOG) performance status score of ≤ 2.
- Adequate organ function laboratory values.
  o Bilirubin ≤ 3 times upper limit range [exception isolated indirect hyperbilirubinemia (Morbus Gilbert-Meulengracht)].
  o Alanine aminotransferase and aminotransferase, ≤ 5 times upper limit range.
  o Creatinine clearance > 30 ml/min (according to Chronic Kidney Disease Epidemiology Collaboration formula).
  o Platelets > 50.000/μl.
  o Absolute CD3^+^ T cell count ≥ 200/μl.
- Negative serological Hepatitis B test or negative PCR in case of positive serological test without evidence of an active infection, negative testing of Hepatitis C RNA, negative HIV test within 6 weeks prior to study inclusion.
- Female patients of childbearing potential and male patients with partners of childbearing potential, who are sexually active, must agree to the use of two effective forms (at least one highly effective method) of contraception. This should be started from the signing of the informed consent and be continued until 3 months (both female and male patients) after last vaccination).
- For female patients of childbearing potential two negative pregnancy tests (sensitivity of at least 25 U/mL) prior to first vaccination, one at screening and the other prior (< 24 h) to first vaccination.
- Postmenopausal or evidence of non-child-bearing status.

Exclusion criteria

- Pregnant or breastfeeding.
- Unwilling or unable to follow the study schedule for any reason.
- Chemotherapy or other systemic therapy or radiotherapy, up to 14 days prior to the first dose of study drug (ongoing low intensity or maintenance treatment with e.g. HMA, venetoclax or midostaurin is permitted).
- Concurrent or previous treatment within 28 days in another interventional clinical trial with an investigational anticancer therapy or any other investigational therapy, which would interfere with the study’s primary and secondary endpoints.
- Major surgery within 28 days of dosing of study drug.
- Treatment with immunotherapy agents within 28 days of dosing of study drug.
- Diagnosis of acute promyelocytic leukemia (APL).
- Autoimmune disease that requires or has required treatment with systemic immunosuppressive treatments, except low dose corticosteroids (< 10 mg/day), in the past 1 year.
- Prior history of malignancies, other than AML/myelodysplastic syndrome (MDS), unless the subject has been free of the disease for ≥ 5 years. Exceptions include the following: basal cell carcinoma of the skin, carcinoma *in situ* of the cervix, carcinoma *in situ* of the breast, histological finding of prostate cancer of TNM stage T1.
- Prior stem cell allograft.
- Ongoing or active infection (including SARS-CoV-2).
- Any live vaccination within 28 days prior to the first dose of the study drug.
- Known hypersensitivity against components of the vaccine.

### 3.2 Study design

**Figure 3:**
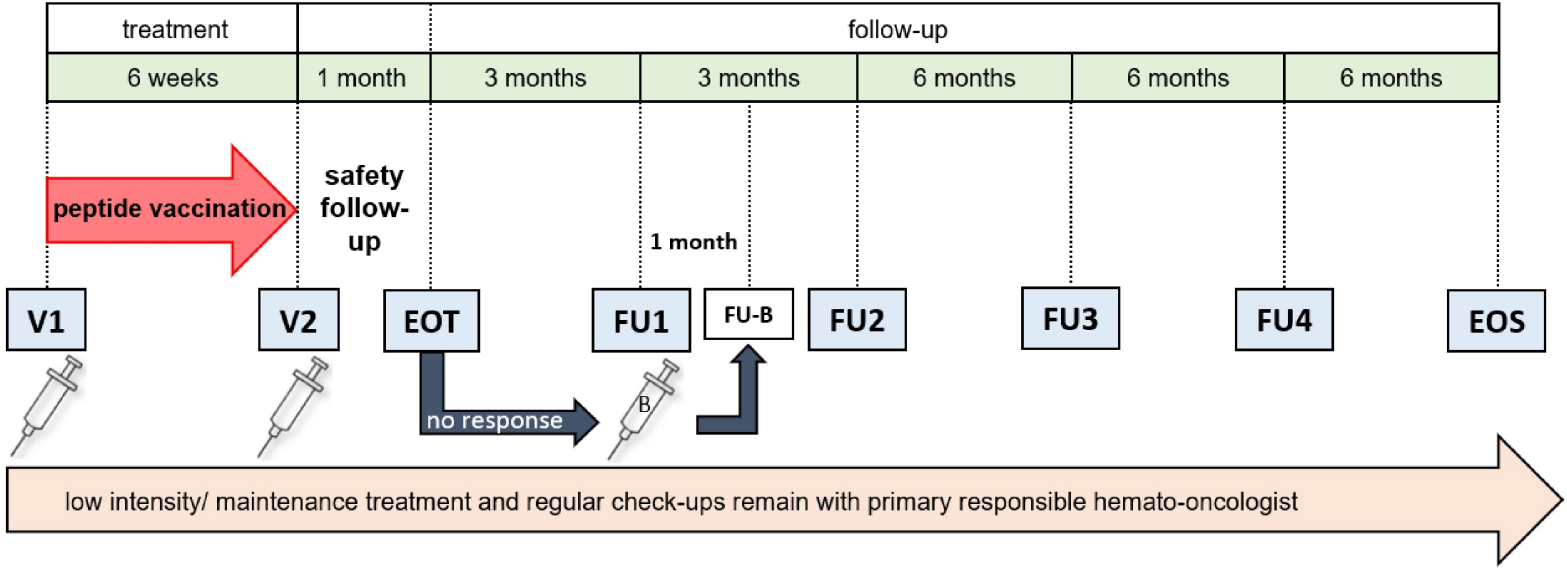
Study schedule. AML patients receive two vaccine doses (V1 and V2), six weeks apart. After one month, an end-of-treatment (EOT) visit is scheduled, followed by two follow-up visits (FU1 and FU2), three months apart. Evaluation of immune response by IFN-γ ELISpot assay is done at every visit except screening. If immune response at EOT is insufficient, a booster vaccination (B) can be applied at FU1, which will then be followed by an additional follow-up visit (FU-B) after one month. After FU2, two more follow-up visits (FU3 and FU4) and an end of study (EOS) visit are performed, spaced six months apart. Throughout the study, any ongoing therapy and its monitoring are conducted by the primary responsible hematologist/oncologist. CR, complete remission; CRi, complete remission with incomplete blood count recovery; n, number.

Trial duration will be approximately 26 months for each patient, however, the actual treatment phase will cover only 1.5 months (**Figure 3**). During that time, patients will receive two vaccine doses of AML-VAC-15, six weeks apart, administered subcutaneously into the skin of the lower abdomen. If an insufficient T cell response is measured four weeks after the second vaccination, a booster vaccination may be applied four months after the second vaccination, which will then be followed up with an extra safety visit four weeks later. This particular dosing scheme is based on results of our previous vaccination trials in healthy adults (P-pVAC-SARS-CoV-2, NCT04546841) (25), cancer patients with B cell/antibody deficiency (B-pVAC-SARS-CoV-2, NCT04954469) (26), glioblastoma patients (GLIO-XS15, NCT04842513) and patients with chronic lymphocytic leukemia (CLL) (XS15-iVAC-CLL01, NCT02802943). Data available from those studies so far shows that the dose of 300 µg per peptide adjuvanted with the TLR1/2 ligand XS15 (50 µg) emulsified 1:1 in Montanide ISA 51 VG is safe and effective to induce profound and long lasting CD4^+^ and CD8^+^ T cell responses that by far exceed those induced by previous peptide vaccines as well as by mRNA-based vaccines (25, 26). Therefore, to balance minimal side effects with maximum immune response induction, two vaccinations spaced six weeks apart are applied within the trial, with the option of a booster for those patients whose T cell responses are still insufficient after two vaccinations.

During the follow-up period, patient care will be supervised by the primary responsible hemato-oncologist center, but patients will report every six months for evaluation of safety, toxicity and primary efficacy, defined as the immunogenicity of the vaccine. Same as during treatment phase, at each scheduled visit the number of AEs according to CTCAE V 5.0 will be recorded and the induction of a T cell response will be determined by IFNγ ELISpot.

### 3.3 Safety and toxicity

Results of repeated safety assessments performed from screening through EOS at scheduled intervals throughout the trial, recruitment rates and trial status will be presented to an independent data and safety monitoring board (DSMB), comprising independent experts in the field of hematology, oncology and immunology. They will evaluate progress, safety data and critical efficacy endpoints in meetings taking place once a year. An extraordinary meeting may be arranged at any time if patient safety appears to be jeopardized (for example, in case a vaccine-related SAE occurs). Thus, the DSMB will regularly assess whether any stopping rules defined in the protocol have been reached and will formulate recommendations for the sponsor on whether to modify, continue or terminate the trial. General stopping rules in this case are an unacceptable profile or incidence rate of adverse events revealed in this or any other study in which at least one of the investigational products of this trial is administered (predefined as the occurrence of more than three vaccine-related SAEs), a demonstration that the study treatment is ineffective or insufficiently active, a significant number of deaths associated with the study treatment and any other factor that in the view of the sponsor constitutes an adequate reason for terminating the study as a whole.

### 3.4 Statistical considerations

According to usual practice in early phase I studies, statistical planning is designed so that a statistically reasoned decision regarding a subsequent phase II study (for or against) can be made. The trial’s sample size was chosen based on the assumption that, in case of the induction of a peptide-specific immune response in ≤ 30% of patients the therapeutic concept will be extended with a probability of at most 5% (type one error, one-sided). Contrastingly, in case of the induction of a peptide-specific immune response in ≥ 60% of patients, the therapeutic concept should be followed with a probability of at least 80% (power). Given a sample size of n = 20 patients, this entails that at least 10 patients must show an immune response for the therapeutic concept to be evaluated in a phase II study. The exact power is 87%, the exact type 1 error is 4.8% (with calculations based on the binomial distribution with n = 20, p = 0.3 or p = 0.6, k < 10 or k ≥ 10).

## 4 Objectives and endpoints of the study

One primary objective of the trial is to evaluate the safety and tolerability of the multi-peptide vaccine AML-VAC-XS15, as determined by the number of adverse events (AEs) according to the common terminology criteria for adverse events (CTCAE) V 5.0. The second primary objective of the trial is to evaluate the efficacy, defined as the immunogenicity, of AML-VAC-XS15 as determined by induction of T cell immunity. This will be evaluated by the percentage of patients with induction of a peptide specific T cell response until 28 days after last vaccination compared to baseline before first vaccination by interferon (IFN) γ enzyme-linked immunosorbent spot (ELISPot), using T cells from peripheral blood.

Secondary objectives of this trial include the induction of peptide specific T cell responses until end of study (EOS), as well as safety and toxicity of the peptide vaccine until EOS. For this purpose, the percentage of patients with induction of a peptide specific T cell response at all scheduled visits after vaccination will be calculated, using T cells from peripheral blood, and the number of patients receiving a booster vaccination will also be documented. Incidence and severity of AEs, serious AEs (SAEs) and suspected unexpected severe adverse reactions (SUSARs) will be documented from first vaccination until EOS. Additional secondary objectives include evaluation of first signs of clinical efficacy, determined by progression-free (PFS) and overall survival (OS) defined as the time from baseline (prior to first vaccination) to the first objective leukemia progression, allogeneic stem cell transplantation or death from any cause until EOS. Finally, secondary objectives include quality of life, analyzed by overall Quality of life scores (EORTC QLC C-30) at all visits during and after vaccination.

Exploratory objectives of the trial are to correlate the inducibility of AML-VAC-XS15 immune responses with clinical, biological, and immunopeptidome-based patient characteristics, as well as a further characterization of the phenotype and functionality of vaccine-induced T cells, which should also enable detection of possible T cell exhaustion. Induction of T cell responses will be determined by IFN-γ ELISpot assays at regular timepoints throughout the trial, compared to baseline and correlated with patient characteristics, as assessed at baseline through to the EOS visit. The vaccine-induced T cell response will be characterized by phenotype and functionality analysis of peptide-specific T cells using flow cytometry and single cell sequencing. These analyses will be correlated to alterations in the number and percentage of lymphocyte subset counts from baseline through EOS. Specific analyses of bone marrow-derived T cells will be conducted as well within expanded exploratory endpoints.

## 5 Discussion

Despite numerous treatment advances for AML in recent years and initially high remission rates, disease relapses occur in a large proportion of patients (1–3). The main reason for disease relapse is the presence of MRD mediated by therapy-resistant LPCs in the patient’s bone marrow (1, 3, 4, 7). Therefore, current efforts are focused on eliminating these cells to achieve long-lasting remissions and potential cures. A rational and promising approach to reach this goal is peptide-based immunotherapy, which represents a low side-effect treatment relying on specific immune recognition of HLA-presented peptides on the tumor cell surface (17, 18). Several peptide vaccination studies have reported promising results in terms of *in vivo* immunogenicity, but so far lack broad clinical responses (30–37). This is likely due to several so far unmet prerequisites for clinical effective peptide vaccination, including the selection of antigens, adjuvant formulations, combinatorial treatments and vaccination time points (18). We present here a first-in-human phase I, single-arm, open-label, interventional multi-peptide vaccination trial that aims to overcome these former limitations by addressing several key prerequisites for clinical effective vaccine design:

Based on our recent work characterizing the immunopeptidomic landscape of LPC in primary AML (23), we designed an AML/LPC peptide vaccine, comprising nine mutated and non-mutated AML- and AML/LPC-associated HLA class I- and HLA class II-restricted peptides. The non-mutated vaccine peptides were selected based on their high frequent natural presentation on AML blasts and particularly on LPCs in a large primary AML cohort. Furthermore, they were validated to be tumor-exclusive, as they were never detected on any benign tissue in an extensive benign immunopeptidome reference database (23). The mutated vaccine peptides comprise two HLA class I-restricted neoepitopes derived from a frameshift insertion in NPM1, which is the most common AML-specific mutation occurring in 35% of all AML patients (38), as well as an HLA class II-restricted neoepitope derived from the AML-specific mutation R140Q in IDH2. In stark contrast to previously conducted neoepitope-based peptide vaccination trials, in which the selection of vaccinated neoepitopes solely relies on *in silico* predictions (39, 40), the neoepitopes included in this vaccine were shown to be naturally presented on AML cells by mass spectrometry-based immunopeptidome analysis and to be targeted by memory T cell responses in AML patients. Of note, for both mutated and non-mutated AML antigens a correlation of HLA peptide presentation as well as peptide-specific memory T cell responses in AML patients with disease outcome was shown, which underscores the pathophysiological relevance of these antigens (23).

The AML-VAC-XS15 vaccine constitutes an off-the-shelf peptide vaccine mainly composed of HLA class II-restricted peptides with promiscuous binding to multiple HLA class II allotypes. The high promiscuity of HLA class II molecules enables the binding to and presentation of the vaccinated peptides by different HLA allotypes and therefore the HLA allotype-independent application of these peptides (41). The feasibility of this approach was recently proven in the P-pVAC-SARS-CoV-2 (NCT04546841) and B-pVAC-SARS-CoV-2 (NCT04954469) trials where 100% and 86% of participants developed a vaccine-induced peptide-specific T cell response independent of their HLA allotypes (25, 26). *In silico* binding predictions demonstrate that the HLA class II-restricted peptides included in the AML-VAC-XS15 vaccine should be able to bind to multiple HLA class II allotypes, which will enable universal patient coverage, in contrast to other approaches employing vaccines limited to particular HLA allotypes (42–44).

Beside the selection of optimal antigen targets, the use of a potent adjuvant formulation able to induce strong and long-lasting immune responses is a key prerequisite for efficient peptide vaccination. In the AML-VAC-XS15-01 study we apply an innovative immune stimulator adjuvant formulation based on the TLR1/2 agonist XS15 that has already proven safety and tolerability as well as potent T cell activation in clinical trials [NCT04546841 (25), NCT04954469 (26)]. XS15, a water-soluble derivative of the TLR1/2 ligand Pam3Cys (24), is combined with the peptide cocktail and emulsified with Montanide ISA 51 VG, resulting in an oily vaccine formulation for subcutaneous injection into the abdominal tissue. This formulation is designed to provide continuous immune stimulation without systemic side effects and has been shown to induce highly potent CD8^+^ and Th1 CD4^+^ T cell responses after single dosing, which exceed those elicited by other peptide and mRNA-based vaccines (25). In addition, the specific adjuvant formulation creates a depot at the injection site where the vaccinated peptides persist, thereby facilitating the induction of a robust immune response lasting up to several years (24–26, 45, 46). Importantly, so far no significant systemic side effects have been reported in healthy volunteers (NCT04546841) (25) or in cancer patients (46), in particular no allergic or anaphylactic reactions or immune-related side effects have occurred as yet.

Peptide vaccination will be applied in AML patients who have achieved CR with first-line therapy. This remission stage ensures an optimal effector-to-target cell ratio for peptide-based immunotherapy, as most of the tumor cells are eliminated and the T cell compartment should be recovered (47–49). Vaccination will start up to 20 weeks after completion of an intensive previous therapy. This time frame will give patients enough time to recuperate the immune system from first-line therapy. With patients receiving maintenance therapy due to an intermediate or high-risk situation, the shortest gap possible between reaching CR and the start of vaccination will be aimed for to minimize the risk of relapse while undergoing vaccination. Patients receiving continuous low-intensity therapy may enter the study as soon as CR/CRi has been achieved.

In summary, the AML-VAC-XS15 vaccine to be evaluated in this study promises to be an innovative treatment to prevent MRD-driven relapse in AML patients who have reached CR with standard therapy. For this particular patient population, which despite initial successes still faces a dismal prognosis, it is expected that the trial will demonstrate preliminary clinical efficacy of an effective, safe and easily applicable new therapeutic option.

## 6 Ethics and dissemination

The clinical trial will be performed in accordance with the Declaration of Helsinki and will comply with the International Conference on Harmonization and Good Clinical Practice. In particular, no study examinations, treatments or procedures will be undertaken without first obtaining written informed consent from the patients. The trial was approved by the Ethics Committee of the Bavarian state medical association and the Paul-Ehrlich-Institut (P01392). During trial conduct, the responsible authorities will be informed on a regular basis about the progress of the trial.

The results of this clinical trial will be presented at relevant national and international meetings and published in peer-reviewed journals regardless of outcome. Written informed consent for publication will be obtained from patients as part of the informed consent form. All planned publications will be reviewed by the principal investigator and the biostatistician prior to publication to avoid violation of patients’ rights.

## Data Availability

The original contributions presented in the study are included in the article/supplementary material. Further inquiries can be directed to the corresponding author.
